# Draft Genome Sequence of the Naturally Competent *Bacillus simplex* Strain WY10

**DOI:** 10.1128/genomeA.01295-17

**Published:** 2017-11-16

**Authors:** Eric C. Keen, Valery V. Bliskovsky, Sankar L. Adhya, Gautam Dantas

**Affiliations:** a The Edison Family Center for Genome Sciences & Systems Biology, Washington University School of Medicine, St. Louis, Missouri, USA; b CCR Genomics Core, National Cancer Institute, National Institutes of Health, Bethesda, Maryland, USA; c Laboratory of Molecular Biology, Center for Cancer Research, National Cancer Institute, National Institutes of Health, Bethesda, Maryland, USA; d Department of Pathology & Immunology, Washington University School of Medicine, St. Louis, Missouri, USA; e Department of Molecular Microbiology, Washington University School of Medicine, St. Louis, Missouri, USA; f Department of Biomedical Engineering, Washington University in St. Louis, St. Louis, Missouri, USA

## Abstract

We sequenced a naturally competent bacterial isolate, WY10, cultured from a Wyoming soil sample. Sequence analysis revealed that WY10 is a novel strain of *Bacillus simplex*. To our knowledge, WY10 is the first *B. simplex* strain to be characterized as naturally competent for DNA uptake by transformation.

## GENOME ANNOUNCEMENT

The Gram-positive bacterial genus *Bacillus* comprises dozens of species and includes obligate human pathogens (*B. anthracis*), facultative human pathogens (*B. cereus*), and nonpathogenic model organisms (*B. subtilis*) (1). Various *Bacillus* species are ubiquitous in soils and other natural environments, and some strains are notable for their natural competence for DNA uptake *in vitro* and *in situ* (2). As part of a previously published study (3), we obtained a bacterial isolate from a soil sample collected in Cheyenne, WY, USA, which formed mucoid off-white colonies on LB plates and spontaneously took up exogenous plasmid DNA in liquid culture. 16S rRNA profiling placed this isolate, then called WY10, within *Bacillus*; here, we report that WY10 constitutes a novel strain of *Bacillus simplex*.

DNA was extracted from a WY10 overnight culture with phenol-chloroform, processed into sequencing libraries (Nextera XT kit; Illumina), and sequenced using 2 × 250 paired-end reads (MiSeq; Illumina). The resulting 4,066,400 paired-end reads were trimmed of Illumina adapters and human-like sequences with Trimmomatic (4) and Deconseq (5), respectively. The remaining 3,924,890 reads (96.5% of original) were assembled with SPAdes (6), yielding a draft genome assembly of 117 contigs (largest contig = 1,148,191 bp; *N*_50_ = 322,734 bp) and 106 scaffolds. All 81 scaffolds >200 bp, comprising 40.3% G+C content across 5,516,951 bp, were functionally annotated with Rapid Annotations using Subsystems Technology (RAST) (7). RAST identified 78 RNAs and 5,739 coding sequences, 44% of which could be functionally categorized. Because individual WY10 scaffolds displayed the greatest sequence similarity by BLASTn to *B. simplex*, JSpeciesWS (8) was used to calculate average nucleotide identity (ANI) based on MUMmer (9) between the WY10 draft genome sequence and all existing *B. simplex* genomes. WY10 shares 93.7% and 90.9% ANI with two completely sequenced *B. simplex* strains, DSM 1321 and SH-B26, respectively, and 85.9% to 97.3% ANI (median ANI = 96.7%) with nine additional *B. simplex* whole-genome shotgun assemblies. Thus, WY10 does not represent a unique type species (conventionally <95% ANI [10]) of *Bacillus* but rather a novel strain of *B. simplex*.

We recently reported that certain bacteriophages, dubbed “superspreaders,” do not efficiently degrade bacterial plasmids during infection and instead release intact plasmid DNA upon lysis, thereby promoting horizontal gene transfer by transformation (3). In this previous work, WY10 was shown to acquire and manifest antibiotic resistance via the uptake of phage-released plasmid DNA. Here, we announce the draft genome sequence of WY10 and its placement within *B. simplex*. To our knowledge, WY10 is the first *B. simplex* strain to be functionally characterized as naturally competent and the first Gram-positive bacterium to demonstrate compatibility with the *Escherichia coli* plasmid pπγ (R6K origin) (11). The draft genome sequence of *B. simplex* WY10 will facilitate further analysis of phage-mediated plasmid transformation and, by extension, horizontal gene transfer and bacterial evolution.

### Accession number(s).

This whole-genome shotgun project has been deposited in DDBJ/ENA/GenBank under the accession number NWQJ00000000. The version described in this paper is the first version, NWQJ01000000.

## References

[1] Bhandari V, Ahmod NZ, Shah HN, Gupta RS 2013 Molecular signatures for *Bacillus* species: demarcation of the *Bacillus subtilis* and *Bacillus cereus* clades in molecular terms and proposal to limit the placement of new species into the genus *Bacillus*. Int J Syst Evol Microbiol 63:2712–2726. doi:10.1099/ijs.0.048488-0. 23475340

[2] Kovács AT, Smits WK, Mirończuk AM, Kuipers OP 2009 Ubiquitous late competence genes in *Bacillus* species indicate the presence of functional DNA uptake machineries. Environ Microbiol 11:1911–1922. doi:10.1111/j.1462-2920.2009.01937.x. 19453701

[3] Keen EC, Bliskovsky VV, Malagon F, Baker JD, Prince JS, Klaus JS, Adhya SL 2017 Novel “superspreader” bacteriophages promote horizontal gene transfer by transformation. mBio 8:e02115-16. doi:10.1128/mBio.02115-16. 28096488 PMC5241400

[4] Bolger AM, Lohse M, Usadel B 2014 Trimmomatic: a flexible trimmer for Illumina sequence data. Bioinformatics 30:2114–2120. doi:10.1093/bioinformatics/btu170. 24695404 PMC4103590

[5] Schmieder R, Edwards R 2011 Fast identification and removal of sequence contamination from genomic and metagenomic datasets. PLoS One 6:e17288. doi:10.1371/journal.pone.0017288. 21408061 PMC3052304

[6] Bankevich A, Nurk S, Antipov D, Gurevich AA, Dvorkin M, Kulikov AS, Lesin VM, Nikolenko SI, Pham S, Prjibelski AD, Pyshkin AV, Sirotkin AV, Vyahhi N, Tesler G, Alekseyev MA, Pevzner PA 2012 SPAdes: a new genome assembly algorithm and its applications to single-cell sequencing. J Comput Biol 19:455–477. doi:10.1089/cmb.2012.0021. 22506599 PMC3342519

[7] Aziz RK, Bartels D, Best AA, DeJongh M, Disz T, Edwards RA, Formsma K, Gerdes S, Glass EM, Kubal M, Meyer F, Olsen GJ, Olson R, Osterman AL, Overbeek RA, McNeil LK, Paarmann D, Paczian T, Parrello B, Pusch GD, Reich C, Stevens R, Vassieva O, Vonstein V, Wilke A, Zagnitko O 2008 The RAST server: rapid annotations using subsystems technology. BMC Genomics 9:75. doi:10.1186/1471-2164-9-75. 18261238 PMC2265698

[8] Richter M, Rosselló-Móra R, Glöckner FO, Peplies J 2016 JSpeciesWS: a web server for prokaryotic species circumscription based on pairwise genome comparison. Bioinformatics 32:929–931. doi:10.1093/bioinformatics/btv681. 26576653 PMC5939971

[9] Kurtz S, Phillippy A, Delcher AL, Smoot M, Shumway M, Antonescu C, Salzberg SL 2004 Versatile and open software for comparing large genomes. Genome Biol 5:R12. doi:10.1186/gb-2004-5-2-r12. 14759262 PMC395750

[10] Richter M, Rosselló-Móra R 2009 Shifting the genomic gold standard for the prokaryotic species definition. Proc Natl Acad Sci U S A 106:19126–19131. doi:10.1073/pnas.0906412106. 19855009 PMC2776425

[11] Srivastava P, Chattoraj DK 2007 Selective chromosome amplification in *Vibrio cholerae*. Mol Microbiol 66:1016–1028. doi:10.1111/j.1365-2958.2007.05973.x. 17944831

